# Supplementary material for: A novel mouse model for checkpoint inhibitor-induced adverse events
Source: PLoS One. 2021 Feb 11;16(2):e0246168. doi: 10.1371/journal.pone.0246168 (PMC7877613; doi:10.1371/journal.pone.0246168)
Supplement: S1 Table — (PDF) [file pone.0246168.s001.pdf]

Supplement Table 1. Basic metabolic panel of mice with multi organ immune infiltration.

Test	Non-Responders	Responders	Normal Range
Total Protein	5.5	6.6	3.5-7.2 g/dl
Albumin	2.65	3.2	2.5-3.4 g/dl
Alk Phosp	47	89	35-96 U/L
Glucose	363.5	404	62-175 mg/dl
Total Bilirubin	0.7	0.6	0-0.9mg/dl
Phosphorus	>15	>15	5.7-9.2 mg/dl
Cholestrol	100	97	40-130 mg/dl
GGT	<10	<10	10-40 mg/dl
ALT	24	35	17-77U/L
Calcium	12.15	13	7.1-10.1 mg/dl
Creatinine	0.2	0.3	0.2-0.9 mg/dl
BUN	28	28.5	8-33 mg/dl
