# Supplementary material for: A novel mouse model for checkpoint inhibitor-induced adverse events
Source: PLoS One. 2021 Feb 11;16(2):e0246168. doi: 10.1371/journal.pone.0246168 (PMC7877613; doi:10.1371/journal.pone.0246168)
Supplement: S1 Data — (PDF) [file pone.0246168.s002.pdf]

Supplement Table 1. Basic metabolic panel of mice with multi organ immune infiltration.

Test	Non-Responders	Responders	Normal Range
Total Protein	5.5	6.6	3.5-7.2 g/dl
Albumin	2.65	3.2	2.5-3.4 g/dl
Alk Phosp	47	89	35-96 U/L
Glucose	363.5	404	62-175 mg/dl
Total Bilirubin	0.7	0.6	0-0.9mg/dl
Phosphorus	>15	>15	5.7-9.2 mg/dl
Cholestrol	100	97	40-130 mg/dl
GGT	<10	<10	10-40 mg/dl
ALT	24	35	17-77U/L
Calcium	12.15	13	7.1-10.1 mg/dl
Creatinine	0.2	0.3	0.2-0.9 mg/dl
BUN	28	28.5	8-33 mg/dl

Minimal data set: Figure 2A

	A	B	C	D	E	F	G	H	I	J	K	L	M	N	O	P	Q	R	S	T	U	V	W	X	Y
1	Mouse number	1	2	3	4	5	6	7	8	9	10	11	12	13	14	15	16	17	18	19	20	21	22	1	3
2	4	38.5*	20.2*	42.6	32.2	28.7	32.7	22.1	32.4*	24.2*	28.6*	46.5*	35.6	37.2*	48.8*	22*	42.9*	28.5	30.3*	30.2*	29.8*	17.3*	29.9*		
3	5	52.3*	34.4*	54	55.7	44.7	37.7	55.3	44.6*	38.5	51.5	70.8	49.4	73	65	51.2	62.1	50.5	65.9	84.5	63.6*	40.4*	53.8*	13.1	33.4
4	6	75.2*	32.4*	100.1	118	45.8	47.4	77.9	78.9*	64.2	62.5	99.1	64.1	80.7	94.1	73.3	85.2	69.3	115.8	77.3	114.3*	68.5*	116*	35.3	46.7
5	7	87.1*	43.4*	96.7	103.9	129.1	81.6	115	89.9*	103.2	89.6	138.6	103.1	104.2	110.1	109	97.3	103.5	114.9	142.4	89.8*	95.2*	80.6*	47.5	60.4
6	8	91.4*	55.8*	78.3	125.7	106.5	116.6	141.2	87.9*	86.8	100.8	102.5	83.6	105.2	95.8	91.4	98.8	109.8	105.8	96.7	102.4*	103.7*	82.7*	61.2	56.9
7	9	69.5*	47.9*	122.3	139.3	171.6	87.4	107.6	86.1*	98.2	104.8	97.7	90.4	146	69.7	93.4	97.6	117.3	129.4	115.4	86*	102.6*	72.6*	80.2	65.7
8	10	94.3*	84.9*	106	131.9	127.3	119	162.7	123.4*	102.5	129.9	149.1	92.1	170.3	130.6	101.4	150.3	137.4	140.3	152.1	112*	108.5*	91*	78.7	65.5
9	11	94.4*	68*	130.2	170.4	204.3	167.6	214.4	136.3*	110.1	130.4	134.8	119.7	179.3	143	152.2	176.8	163.2	204.5	136	119.1*	127.2*	86.4*	90.8	50.3
10	12	82.7*	59.3*	126.4	314.8	163.7	193.3	271.5	118.2*	120.4	172.5	160.1	130.7	213.4	156.1	131.2	169.3	150.9	199.8	152	128.3*	130.8*	97.9*	87.8	57.2
11	13	85.1*	44.1*	171.1	305.3	297.7	173.5	180.4	90.8*	128.2	196.2	173.1	106.7	221.9	159	123.2	178.9	194.5	244.6	215.2	126.9*	144.1*	94.7*	107.2	39.4
12	15	68.4*	77.7*	145.6	558	342.5	136.7	328.3	80.3*	125.3	257.9	222.1	109.6	271.9	211.8	83.1	268.7	215.8	337.2	254.4	78.7*	109.3*	147.6*	116	47.8
13	17	92.7*	99.5*	238.1	616.4	457.1	129.8	234.1	72.5*	150.6	304.1	313.8	81.3	356.1	225.2	34	364	278.7	380.8	281.5	36.7*	124.9*	81.9*	127.5	67
14	20	116.9*	105.3*	262.9	1123.9	589.1	248.2	491.5	75.3*	208.1	454	479.5	65.5	487.9	282	21.2	416.9	397.4	550.4	338.1	6.2*	97.2*	81.1*	229.9	51.8
15	22	124.9*	147.7*	329.2	1060.2	736.9	271.6	557.5	59*	444.1	448.2	548.3	51.7	643.1	399.6	8.5	521.9	445.5	634.3	487.7	7.2*	124.9*	69.9*	271.7	109.6
16	24	218.8*	265.4*	361.4	1411.4	886.5	648	870.3	75*	350	795.5	726.7	94.2	732.5	492	0	757.3	551.6	626.5	605.7	0*	178.9*	57.4*	337.5	141.6
17	27	406.8*	339.9*			1241.9	1208.3	1243.6	153.1*		993.8	1037.5	133.3	1058.4	711.8	0	1073.1	779.8		932.9	0*	482.1*	69.8*	604.5	164.8
18	28	415.3*	321.6*			1471	1416.6	1334.2	157.3*		944.5	1059.7	120.9	1180.1	697.8	0	1195.8	825.8		950.5	0*	583.6*	67.1*	657.1	208.1
19	29	577.6*	667.9*			2118.9	1726.3	1444.5	282.5*		1347.8	1295.1	202.3			0		966.6			0*		99.6*	703.1	229.9
20	30*	743.8*	741.2*			2183.6*	2274.2*	1806.4*	286.4*		1326.9*	1448.9*	259.3*			0*		952.2*			0*		115.7*	650.9*	204.6*
21	36*	1416.5*	1736.3*					3192.8*	630.9*		1966.6*	2224.3*	554.9*			0*		1371.6*			0*		222.2*	1706.1*	289.1*

Minimal data set: Figure 2B

	A	B	C	D	E	F	G
1	Days	Responders			Nonresponders		
2	-10	22	19		18	19	20
3	-2	23	20		19	20	19
4	1	21	19		19	18	20
5	6	22	21		20	20	21
6	13	22	20		20	19	21
7	19	23	22		20	20	22
8	26	22	24		23	21	22

Minimal data set: Figure 3B

Days	Untreated (juts for reference)	PD1/CTLA4/Prednisolone			PD-1/CTLA4 (Responders)			PD-1/CTLA4 (Non-Responders)			
-3	*13.1**	75.2	32.4	78.9	64.1	73.3	33.4	62.5	99.1	77.9	69.3
-2	*35.3**	87.1	43.4	89.9	103.1	109	46.7	89.6	138.6	115	103.5
-1	*47.5**	91.4	55.8	87.9	83.6	91.4	60.4	100.8	102.5	141.2	109.8
0	*61.2**	69.5	47.9	86.1	90.4	93.4	56.9	104.8	97.7	107.6	117.3
1	*80.2**	94.3	84.9	123.4	92.1	101.4	65.7	129.9	149.1	162.7	137.4
2	*78.7**	94.4	68	136.3	119.7	152.2	65.5	130.4	134.8	214.4	163.2
3		82.7	59.3	118.2	130.7	131.2		172.5	160.1	271.5	150.9
4	*90.8**	85.1	44.1	90.8	106.7	123.2	50.3	196.2	173.1	180.4	194.5
5	*87.8**						57.2				
6	*107.2**	68.4	77.7	80.3	109.6	83.1	39.4	257.9	222.1	328.3	215.8
7	*116**						47.8				
8	*133**	92.7	99.5	72.5	81.3	34	47.6	304.1	313.8	234.1	278.7
9	*127.5**						67				
10											
11	*169.7**	116.9	105.3	75.3	65.5	21.2	77	454	479.5	491.5	397.4
12	*229.9**						51.8				
13	*242.9**	124.9	147.7	59	51.7	8.5	89.6	448.2	548.3	557.5	445.5
14	*271.7**						109.6				
15	*287.1**	218.8	265.4	75	94.2	0	137.2	795.5	726.7	870.3	551.6
16	*337.5**						141.6				
17	*494.2**						146.7				
18	*500.6**	406.8	339.9	153.1	133.3	0	147.7	993.8	1037.5	1243.6	779.8
19	*604.5**	415.3	321.6	157.3	120.9	0	164.8	944.5	1059.7	1334.2	825.8
20	*657.1**	577.6	667.9	282.5	202.3	0	208.1	1347.8	1295.1	1444.5	966.6
21	*703.1**	743.8	741.2	286.4	259.3	0	229.9	1326.9	1448.9	1806.4	952.2
28	*1706.1**	1416.5	1736.3	630.9	554.9	0	289.1	1966.6	2224.3	3192.8	1371.6
